# Development of a Three-Dimensional Hand Model Using Three-Dimensional Stereophotogrammetry: Assessment of Image Reproducibility

**DOI:** 10.1371/journal.pone.0136710

**Published:** 2015-09-14

**Authors:** Inge A. Hoevenaren, J. Meulstee, E. Krikken, S. J. Bergé, D. J. O. Ulrich, Thomas J. J. Maal

**Affiliations:** 1 Department of Plastic, Reconstructive and Hand Surgery, Radboud University Nijmegen Medical Centre, Nijmegen, the Netherlands; 2 Department of Oral and Maxillofacial Surgery, Radboud University Nijmegen Medical Centre, Nijmegen, the Netherlands; 3 Technical medicine, University of Twente, Enschede, the Netherlands; University of Nebraska Medical Center, UNITED STATES

## Abstract

**Purpose:**

Using three-dimensional (3D) stereophotogrammetry precise images and reconstructions of the human body can be produced. Over the last few years, this technique is mainly being developed in the field of maxillofacial reconstructive surgery, creating fusion images with computed tomography (CT) data for precise planning and prediction of treatment outcome. Though, in hand surgery 3D stereophotogrammetry is not yet being used in clinical settings.

**Methods:**

A total of 34 three-dimensional hand photographs were analyzed to investigate the reproducibility. For every individual, 3D photographs were captured at two different time points (baseline T0 and one week later T1). Using two different registration methods, the reproducibility of the methods was analyzed. Furthermore, the differences between 3D photos of men and women were compared in a distance map as a first clinical pilot testing our registration method.

**Results:**

The absolute mean registration error for the complete hand was 1.46 mm. This reduced to an error of 0.56 mm isolating the region to the palm of the hand. When comparing hands of both sexes, it was seen that the male hand was larger (broader base and longer fingers) than the female hand.

**Conclusions:**

This study shows that 3D stereophotogrammetry can produce reproducible images of the hand without harmful side effects for the patient, so proving to be a reliable method for soft tissue analysis. Its potential use in everyday practice of hand surgery needs to be further explored.

## Introduction

Nowadays, three-dimensional (3D) surface imaging is being implemented in various healthcare and non-healthcare areas, such as anthropology, reconstructive surgery, craniofacial surgery and orthodontics. One of the surface imaging techniques being used more and more is 3D stereophotogrammetry [1,2]. Within the broad field of reconstructive surgery, it has already found solid applications in maxillofacial surgery [3–6] and is upcoming in breast and free flap reconstructive surgery [5,7–11]. However, 3D imaging of the hand is an unexplored field so far. The possibilities of this technique in the clinical and pre-clinical setting are numerous, for example using it for preoperative planning or postoperative evaluation of hand surgeries, and for various educational purposes.

The aim of this study was to develop a noninvasive, objective and valid method of photographing the hand using 3D stereophotogrammetry. Thereafter, making use of the standardized 3D images, we started evaluation of the reproducibility and validity of newly defined soft tissue landmarks of the hand for clinical purposes, by using our registration method on images of the hands of men and women.

## Subjects and Methods

### Subjects

**Fig 1:**
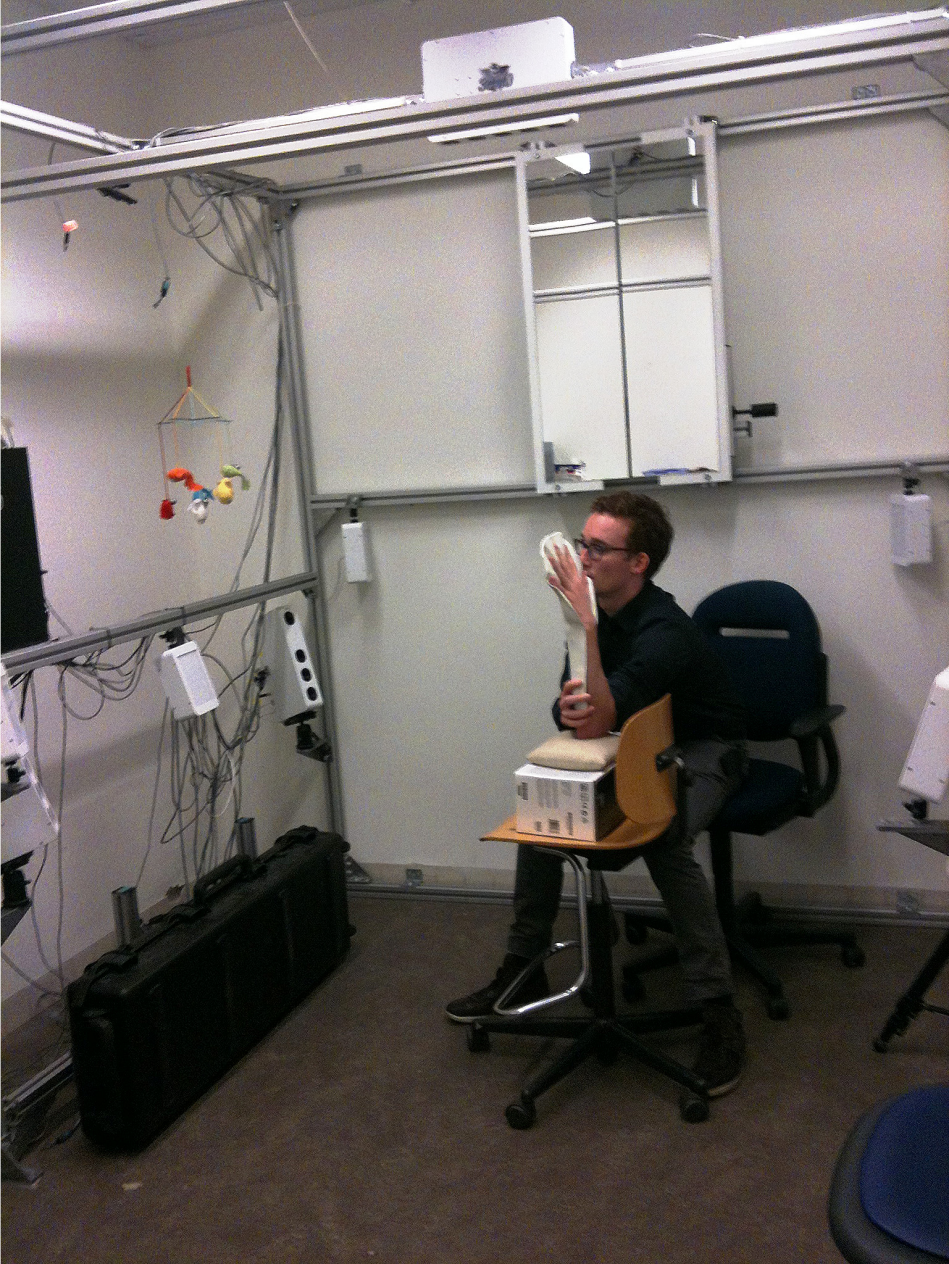
3D stereophotogrammetrical camera alignment. Example of the three-dimensional stereophotogrammetrical camera alignment.

Seventeen adult Caucasian individuals (4 male, 13 female; mean age 39.1 years, range 26–64 years) without any preexistent hand deformities were randomly selected from our departments. All individuals gave written informed consent to participate in this study. Approval by a medical ethics review board was not obtained, since the digital photography method used was previously described by the review board as not invasive or harmful. This study was conducted in compliance with the World Medical Association Declaration of Helsinki on medical research ethics. 3D photographs of both the left and right hand were taken, resulting in a total of 34 images. The photographs were taken with the subject positioned as shown in Fig 1. The individual in this figure has given written informed consent (as outlined in PLOS consent form) to publish these case details.

### Imaging methods

For every individual, 3D photographs were captured at two different time points (baseline T0 and one week later T1). The 3D photographs were captured with a 3D stereophotogrammetrical camera setup and the software program Modular System v1.0 (3dMDface System, 3dMD, Atlanta, USA). The camera setup consisted of five pods, each equipped with three digital cameras and a flash. Prior to its use, the camera was calibrated to define a 3D coordinate system for the 3D photograph, which was referred to as the original 3D coordinate system [12,13]. The 3D photographs were taken with each individual seated with the arm in a 90 degree flexion in the elbow joint and the hand kept in a pre-designed template with the fingers in a fully spreaded, upright position (Fig 1). In order to achieve exact images of both the palmar and dorsal aspect of the hand, the template was removed by an assistant, directly before the photograph was taken. The patient was instructed not to move the hand to another position and to keep it absolutely still after removal of the template.

### Reproducibility of the 3D photographs

The 3D photographs of the left and right hand of the same individual acquired at different time points were registered using the Iterative Closest Point (rigid registration) algorithm [14]. After registration the difference between the two surfaces was calculated as an error or distance map indicating the amount of similarity between the registered surfaces. This distance map computes the difference (Euclidean distance) between the 3D photographs on a large number of points and is an objective measure for the reproducibility of the acquired 3D photographs. The absolute mean value as well as the 50th and 90th percentile of the registration error were computed in millimeters.

Apart from the distance map of the complete 3D model of the hand, a distance map was calculated of only the palm of the hand. Hereby it was possible to investigate whether the palm is a more rigid region compared to the individual fingers which have more variables of free movement. For the distance map of the palm of the hand, the 50th and 90th percentiles were computed.

### Landmarks and registration

After investigating the reproducibility of the image acquisition method, anatomical landmarks were placed on each of the 34 photographs according to the method developed by Hoevenaren et al. [15]. These landmarks could then be used for comparing different hands and to compute an average hand model. In order to compare different hands and compute an average hand, the landmark sets of all 17 left hands and 17 right hands were registered using two different registration methods.

#### Method 1

The first registration method was a general Procrustes registration of the complete landmark set of either the left or right hand images. At first, one of the landmark sets is randomly selected as a reference set. Towards this set, all other landmark sets are registered by matching the similar landmarks in each set. After completing this registration an average hand surface model can be computed. Thereafter, this complete process of matching similar landmarks of each set is repeated, now using the newly created average hand as reference set for the registration procedure. After complete registration, a new average hand surface model is computed.

#### Method 2

The second method uses an adapted version of the Iterative Closest Point algorithm in which the hand is divided into different anatomical sub regions namely the palm of the hand, the thumb, the index finger, middle finger, ring finger and the little finger. All different sets were then registered with a randomly selected reference set, resulting in a computed, average hand. Also in this method, the complete procedure was repeated with the average hand performing as the reference set (see method 1).

For both registration methods the RMS error was calculated as a measure of precision of the computed model. The reproducibility of method 1 and method 2 were evaluated using the paired t-test. Statistical analyses were performed with the IBM SPSS software program, version 20.0 for Windows (IBM Corp., Armonk, NY, USA). The level of significance for all statistical analyses was set at 5%.

### Comparison of Male and Female Hands

Using the described registration methods, it was now possible to register the hands of different subjects in a clinical setting. As a pilot study, the average hand of five male and five female subjects were calculated and compared.

## Results

### Reproducibility

**Fig 2:**
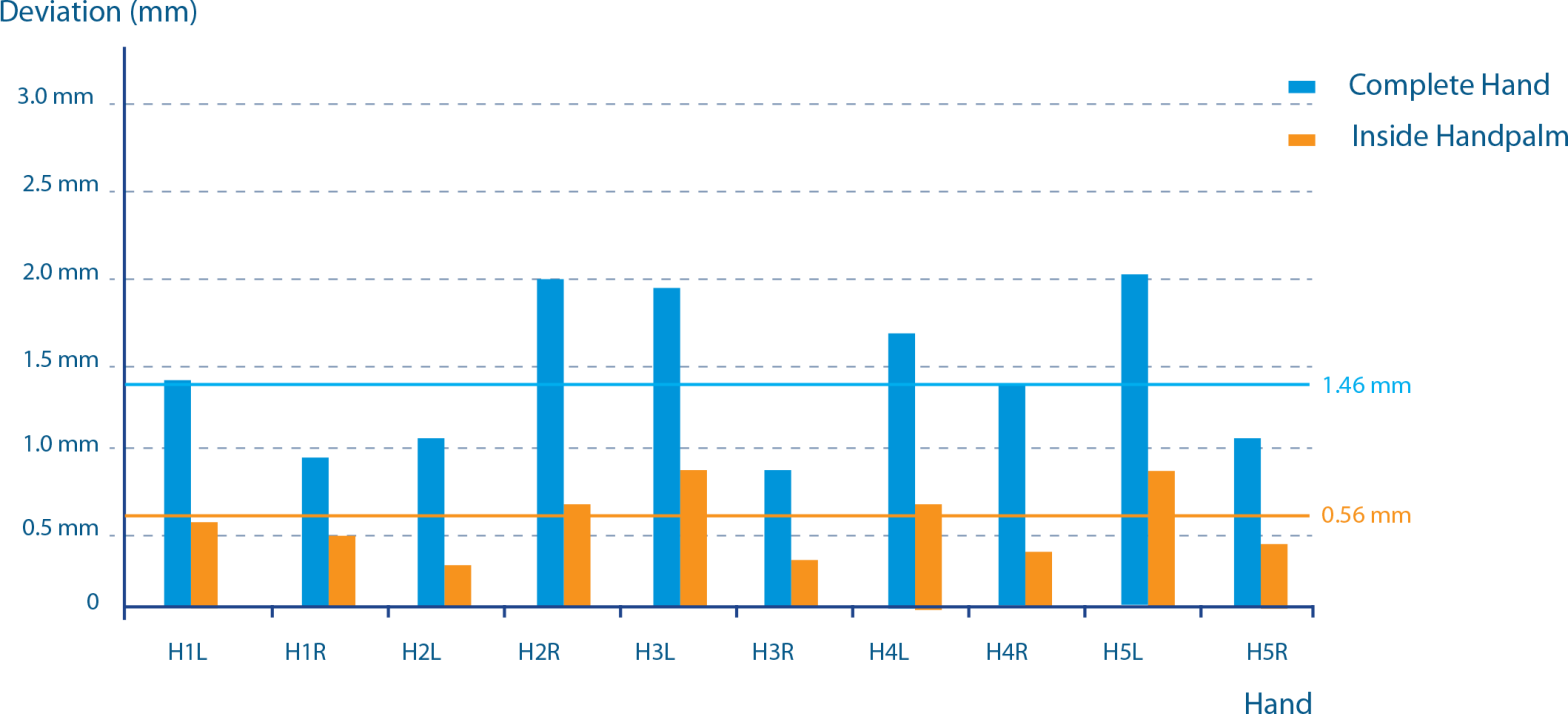
Registration errors. The registration errors indicating the reproducibility of the image acquisition method are illustrated here. Reproducibility of 3D images with deviation on vertical axes in millimeters and on horizontal axes the measured hands. H1L is the first left hand, H1R is the first righ hand, etcetera. The mean deviations of registration on the complete hand and on the palm of the hand are respectively 1,46 mm and 0,56 mm.

The absolute mean registration error for the complete hand was 1.37 mm. The 50th and 90th percentile of the registration error were respectively 0.97 mm and 2.95 mm for the complete set of hands. Isolating the region to the inside of the hand, the absolute mean error was reduced to 0.56 mm. The 50th and 90th percentile of the registration error were respectively 0.47 mm and 1.11 mm for the isolated region of the hand palm. The registration errors indicating the reproducibility of the image acquisition method are illustrated in Fig 2.

### Calculation of average hands

**Fig 3:**
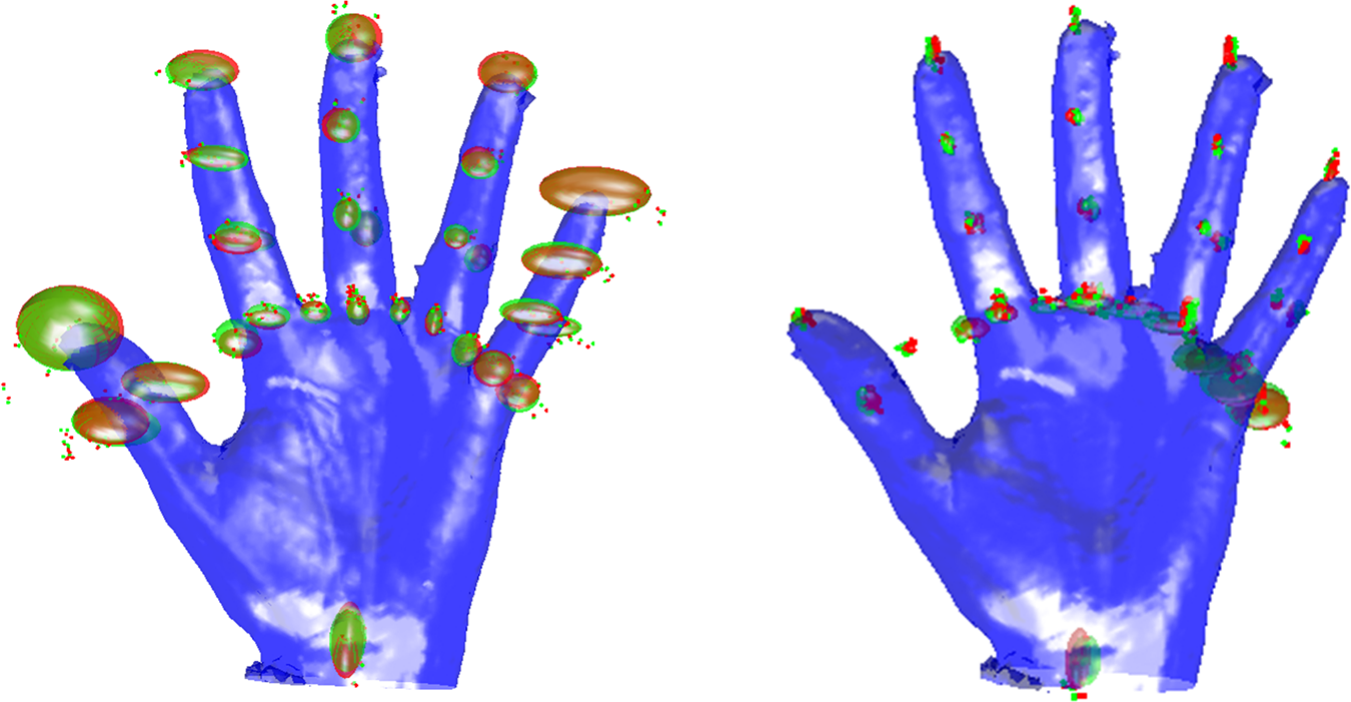
Landmarks and ellipsoids. Comparison of General method and Hands method. Illustrated are the average landmark set and ellipsoids which are calculated as two times the standard deviation.

For the registration of method 1 a RMS error of 0.94 mm was found. For method 2, in which the fingers were registered as separate models the RMS error decreased to 0.42 mm. The variation of registered landmark sets could also be illustrated. Fig 3 illustrates the average landmark set and ellipsoids which are calculated as two times the standard deviation.

### Comparison between male and female hands

**Fig 4:**
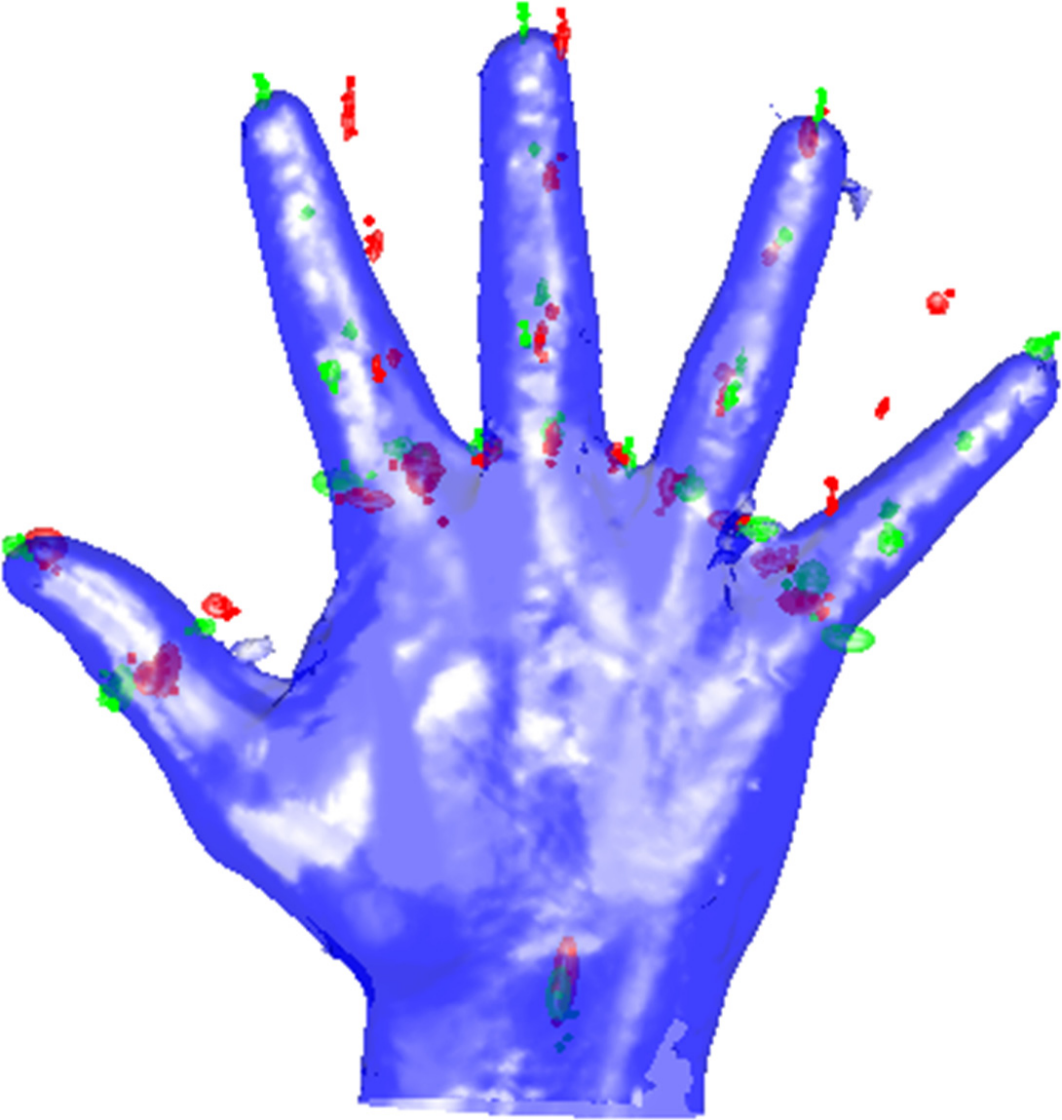
Comparison of male and female hands. Illustration of the differences between the male (in green) and female (in red) average hands.

The male and female hands were compared using method 2. An illustration of the difference between the male and female average hands can be found in Fig 4. In general the male hand is larger (broader base and longer fingers) than the female hand.

## Discussion

With the advent of digital technology, digital photography has become an increasingly important tool in reconstructive surgery, in particular in maxillofacial surgery [16]. With the introduction of systems such as the 3dMDface System (3dMD LLC, Atlanta, USA), Di3D (Dimensional Imaging, Glasgow, UK) and 3D-Sensors FaceSCAN3D (3D Shape GmbH, Erlangen, Germany), the applicability of 3D photography in daily practice has become reality. 3D stereophotogrammetry is safe, noninvasive and able to capture superior quality ‘external surface’ 3D photographs in less than 10 milliseconds [17]. Especially these characteristics are ideal to collect 3D data even of hands of young children or elderly patients. After processing the data, an exact digital model of the patients hand is created which can be used in a clinical setting immediately.

In the complex field of hand surgery, 3D photography is not yet being used in clinical settings as far as we know. However, the hand is a complex anatomic part of the body and its injuries are very incapacitating for the patient, making precise planning of every hand surgery procedure and predicting its outcome necessary in obtaining a satisfying postoperative result for both patient and doctor.

### 3D imaging

At present, the gold standard in imaging soft and bony tissues of the hand are computed tomography (CT) data or magnetic resonance imaging (MRI) scan images with their known side effects [18–20]. However, these techniques do not produce a real-life image of the outer surface of the hand. The shortcomings of CT and MRI scan images have caused a search for a minimally invasive 3D imaging technique, which could produce a realistic and exact virtual surface model of the hand. Among others, advantages of 3D stereophotogrammetry over MRI and CT are its non-invasiveness, lack of harmful radiation, immediate available images and good patient tolerance [5]. Furthermore, the usage of 3D stereophotogrammetry is highly cost effective, given the very short amount of time needed for photographing and low production costs, compared to the time consuming CT or MRI scan images. Besides the absence of potential dangerous side effects described above, there is the possibility of patient-specific modeling. 3D imaging has the potential to precisely simulate the pre-operative experience for surgery planning with reliable surface visualization leading to a more profound understanding of the anatomical structures to be reconstructed and therefore improved final surgery outcome.

The aim of this study was to develop a noninvasive, objective and valid method of photographing the hand using 3D stereophotogrammetry. Furthermore, we evaluated the reproducibility and possibility of computing an average hand and using it in a clinical pilot.

### 3D stereophotogrammetry

A review of literature showed there is no information on the reproducibility and validity of 3D hand photography yet. Earlier studies were performed to investigate the precision of 3D stereophotogrammetry. The majority of these studies focused on reliably measuring distances between typical anthropometric points on the 3D reconstructed images against corresponding points on live subjects or phantom models (e.g. plaster casts) as a form of validation [12,21–26]. Some other studies use more complex methods to obtain and analyze 3D shapes [27–29].

Kau et al., Maal et al. and Ma et al. investigated the reproducibility of several 3D acquisition systems. Kau et al. found an RMS error of 0.4 mm using a Minolta Vivid 900 laser scanner [30]. Maal et al. used 3D stereophotogrammetry, assessed the reproducibility and also found a value of 0.4 mm [31]. Ma et al. found a reproducibility of their structered light system of 0.2 mm [22].

### Image reproducibility

In this study two different registration methods were used, method 1 matching complete hands and method 2 matching subregions of the hand. We found a significant higher registration error for method 1. An explanation for this could be found in the fact that it was difficult for the study subjects to keep their fingers completely still at time of photography. Therefore, we designed method 2, correcting for the movement of the individual fingers.

### Average hand model

Using method 2 as valid registration method, an average model of both left and right hand 3D images was created. This method of landmark registration and matching has already found solid applications in maxillofacial surgery. In this study, as stated above, one of the difficulties in creating reproducible images of the hand is the movement of the individual fingers. However, we corrected this error by designing a new method of landmark based registration, matching each individual finger per image and therefore creating a more precise image.

In maxillofacial surgery ‘the average face’ is an instrument often used to which other facial images are being compared [32,33]. It is mainly being used in comparing preoperative images of patients and predicting treatment outcome, thereby reducing operating time. In literature, no publications exist on the average hand. Given the proven reproducibility and clinical value of calculations based on the average face, we developed a method of creating an average hand model. One of the drawbacks of this study is the small population on which this model is based. Therefore, a database of 3D hand images is being collected at the moment, created from study subjects from different age categories.

### Clinical Relevance

The possibilities of the clinical and pre-clinical use of 3D photography of the hand are numerous. 3D images could be used in pre- and postoperative hand trauma surgeries, thereby quantifying the soft tissue loss and identifying the optimal reconstructive surgery type. Furthermore, during follow-up after nerve injuries of the upper extremity, soft tissue volume deficiencies can be calculated, evaluating treatment results and indicating the need for revision surgery. A normative database based on soft tissue data is not yet available, but needed. This database could be used among others in growth studies of both normal child development and pathologic growth, for example in patients with acromegaly, and in objectifying the sex-specific differences of the hand.

Using the landmark based registration method 2, we describe the first clinical pilot with this study. Though a very small population, we confirmed the reliable use of the method calculating differences in hands of both men and women. Further research with a larger study population is necessary to validate the advanced clinical use.

Besides its use in clinical practice, the developed 3D hand model can be used for various educational purposes. Computer-assisted learning has been widely established in medical anatomy learning, since it facilitates obtaining anatomic knowledge in a flexible and efficient way [34–40]. With medical imaging quickly becoming a more cost-effective source for clinical and pre-clinical training, a growing number of 3D models for different anatomic regions are being developed [40–43]. Since 3D photography produces precise images and reconstructions of the human body, these models support comprehension of spatial anatomy in diagnostic and surgical interventions [9,41,42,44,45].

Also, 3D real-life models based on 3D stereophotogrammetry could be used for exact simulation in surgical resident training, increasing the knowledge of the difficult anatomical areas of the human body and therefore improving the quality of teaching [6,39,46].

In addition, there is an application in forensic medicine, where with the aid of a database, skin reconstructions can be created out of osseous parts of the hand for identification purposes [47].

Since the hand is not a static anatomical structure and precise finger movement is one of its main functions, our future research focuses among others on combining 3D techniques with CT data. Recently, the possibility of creating fusion images with CT data is extensively being used for even more precise planning and predicting treatment outcome [9,48,49]. In conclusion, this study shows that 3D stereophotogrammetry produces precise and reproducible 3D images of the hand. This proves 3D photography to be a reliable method for soft tissue analysis. Its potential use in everyday practice of hand surgery and the concept of fusing 3D photography images with radiologic images of the interior hand structures needs to be further explored.
